# Logistics Distribution Route Optimization Based on Genetic Algorithm

**DOI:** 10.1155/2022/8468438

**Published:** 2022-07-06

**Authors:** Liu Xin, Peng Xu, Gu Manyi

**Affiliations:** School of Humanities and Management, Southwest Medical University, Luzhou 646000, China

## Abstract

Aiming at the problem of logistic division based on genetic algorithm, it is planned to study the improvement of logistic distribution methods. We first meet the requirements of the genetic algorithm of logistic development, use the division method to divide the delivery area of the gene, and formulate a functional delivery plan, which generally includes weight measurement, measurement time, customer value measurement, instrument measurement time, and the whole process index. We set weight goals and find the best way to improve genetic algorithm delivery. The experimental comparison results show that the optimal method takes less than 2 minutes to find the optimal method, while the normal process takes 4 minutes to find the optimal method, and the longest can reach 5 minutes. The comparison shows that the traditional algorithm takes longer to find the correct way than the algorithm developed this time. Finally, the simple logistic distribution optimization method model and the soft time-limited logistic distribution processing optimization model are calculated and simulated by the genetic testing algorithm and genetic algorithm development. The effectiveness of the improved genetic algorithm in local research and the effectiveness of the logistic transportation allocation solution are determined.

## 1. Introduction

Due to the increase in the timely delivery of small and large batches of goods, the transportation cost has increased year by year, and the transportation of many merchants has exceeded the sale price. Choosing the best mode of transportation has become an important measure to control logistic costs. The VRP (vehicle routing problem) is a major problem in transportation today and is slowly gaining attention. In this study, an improved genetic algorithm is used to solve the logistic distribution routing problem. The focus of the distribution operation of the logistic center is how to effectively use the vehicle and determine its most economical driving route map, so that the goods can be delivered to the customers in the shortest time. Distribution and transportation are the most important functional elements in logistic and distribution activities, and the rationalization of distribution largely depends on the rationalization of distribution and transportation. The goal of in-store delivery is how to properly use the vehicle and determine its best driving position to deliver the goods to the customer in a short period of time. The functional elements of an express logistic system refer to the basic capabilities of the express logistic system. These basic capabilities are effectively combined and connected, becoming the general function of express logistics, and the overall purpose of the express logistic system can be reasonably and effectively realized. The functional elements of the express logistic system are generally considered to have transportation, storage, packaging, loading and unloading, handling, circulation and processing, distribution, express logistic information, etc. If viewed from the actual work links of express logistic activities to examine, the express logistics is composed of the above seven specific works. In other words, express logistics can achieve the above seven functions. Distribution and shipping are the most important roles in distribution.

Rationalization of distribution depends on the rationalization of distribution and transportation measures. Due to the large number of urban customers and the difficulty of distribution, how to build the best road is not only the characteristics of distribution and transportation, but also an arduous task. Whether the distribution is reasonable or not has a significant impact on export speed, cost, and benefit. Applied research determining product distribution, especially through consumer distribution, is an important and difficult task in product distribution. It is an important way to improve the performance of freight forwarders to understand the research of logistic distribution vehicle routing optimization.

## 2. Literature Review

The logistic industry plays an important role in international trade and plays a leading role, and more importantly, it has a greater impact on all aspects of the global economic influence. By business, transportation, information technology industry, and the development of talent employment industry, we prompted the warehouse storage, logistics, goods processing and distribution, and cargo loading and unloading industry development; formed a relatively complete new industrial chain, optimized the allocation of resources; to a large extent, pulled the domestic demand; also provided an opportunity for youth entrepreneurship; and brought social and economic development and prosperity. Shipping has gradually become an important challenge for enterprises [1]. The goal of export products is to provide customers with the best service at the lowest price, so as to improve the overall efficiency of the industry, increase the overall profit, and improve market competitiveness. Therefore, studies on export improvement are not only concerned with the cost-effectiveness and service efficiency of the logistic industry, but can also provide data for income distribution. Manufacturers maximize efficiency and enhance the overall image of the enterprise.

**Figure 1:**
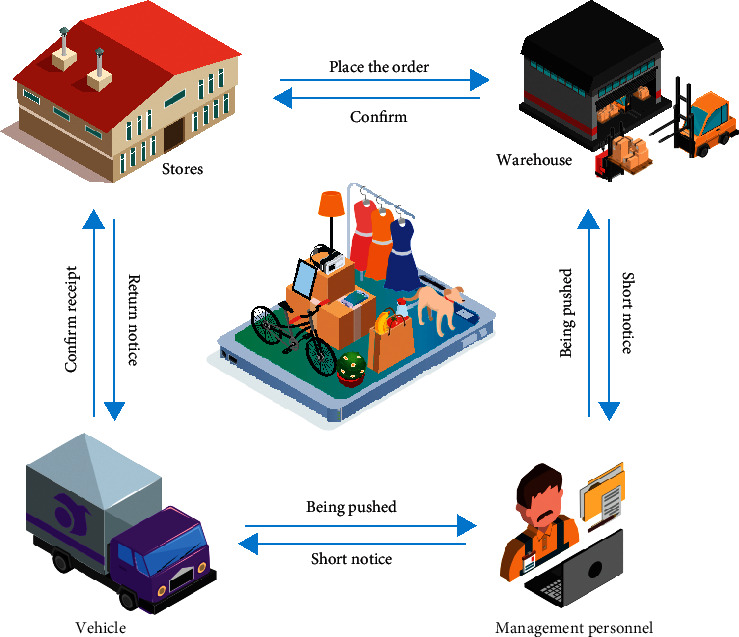
Logistic distribution path of genetic algorithm.

The optimization problem has always been the focus of scientists' research, and the research on the distribution types of joint ventures is in the first stage. Asokan et al. obtained centralized joint ventures and equipped them with multiobjective genetic optimization to solve the split order problem in joint ventures [2]. Wang and Ying used fuzzy programming models and algorithms to consider the uncertainty of vehicle travel and customer service time, developed a collaborative fuzzy programming model with the problem of minimizing vehicle delivery costs, and adopted an adaptive discrete particle swarm optimization algorithm to solve the problem [3]. Based on the concept of virtual business and capital integration, Li et al. provided a model for the integration of third-party logistic companies in a virtual environment and simulated the model with AutoMod simulation software [4]. Ran et al. have developed an integrated circuit system that takes advantage of the point-to-point power distribution model and the hub electrical model to provide research-specific heuristics. Using this method is very effective in emergency situations [5]. Martinson et al. argue that joint ventures can reduce product distribution costs, increase revenue, and improve business efficiency through capital sharing. Whether the joint venture is healthy or not depends on whether the joint venture's cost sharing and distribution of benefits are reasonable. Considering the income and total cost, this study proposes a new concept of income distribution [6]. Daugherty et al. have developed one of the multi-purpose stochastic programming models for transportation emergency construction using a combination of vertical and horizontal distribution systems [7]. The traffic crisis (VRP) was first mentioned by Sun et al. Since then, the VRP has become an important research hotspot and research work in the field of combinatorial optimization [8]. Yang et al. proposed a segmentation method for the VRP problem. To this end, they directly determine the set of best possible solutions (possible solutions) and develop a simple mathematical model of VRPAs, as shown in Figure 1 [9].

Based on the current research, a study is planned for the delivery of the optimization process. First, in order to meet the requirements of the development of genetic algorithm logistics, the logistic distribution path of genetic algorithm is optimized to solve the problem of finding the optimal path. Second, we adopted the clustering algorithm of genetic algorithm logistic distribution area and establish logistic distribution path optimization target function, mainly including weight index, aging index, customer importance index, time window index, and total path index, and set the distribution target weight, to find the optimal target path, in order to realize the genetic algorithm logistic distribution path optimization. For today's enterprises, how to reduce operating costs, increase profits, and improve transportation has become a major issue in logistic management, which directly affects and determines the importance of competition in the logistic industry. On the one hand, the logistic industry is facing good development prospects, and on the other hand, it is also facing a bottleneck restricting its development. Especially with the acceleration of economic globalization and information technology, a series of outdated modes such as traditional and simple transportation and storage services have limited and delayed the generation and development of efficient and professional logistic services. In industrial areas, many logistic centers choose product distribution according to customer needs, resulting in less resource usage and high cost. Sharing can be a new way of distribution. Codistribution is to exchange the previous allocation according to different suppliers and different products into “heavy equipment and allocation” that is no different from suppliers and inventories. That is, transporting goods on the same route, using the same truck to transport multiple customers, reduces operating costs, and improves resource utilization and the competitiveness of the logistic industry.

## 3. Logistic Distribution Path Optimization Based on Genetic Algorithm

### 3.1. Significance of Route Distribution Optimization

From the application point of view, logistic distribution optimization is not only an important part of logistic distribution optimization but also an indispensable topic in e-commerce games. Optimizing freight transport can improve the efficiency of logistic transactions and promote logistic research. The research theory and process of optimizing freight routes are the basis for developing transportation, creating a modern reference command system, developing professional transportation technology, and developing e-commerce. In the construction of logistic distribution information network platform in most rural areas, due to various conditions, there are still many deficiencies in the design of the platform. One is that most of the products sold in rural areas are still sold and circulated in small shops. There are many sectors of wholesale trade, but most involve traditional industries. Farmers generally rely on traditional methods to obtain information on the circulation and distribution of agricultural products, with little access to news or the internet, and the rest from local markets. Traditional practices of raw data, logistics, and distribution are depriving most rural areas of the environment in which information networking platforms are built. Second, while some agricultural discussions focus on product availability, in practice, most sites do not have robust export plans, reasonable standards, or affected export directions. For example, in the data distribution process, the lack of an objective data management interface can lead to data distortion and data expiration. Therefore, the construction of the existing logistic distribution information platform in most rural areas is not perfect [10].

### 3.2. Basic Concepts of Genetic Algorithm

The genetic algorithm (GA) is an intelligent algorithm. This is an important way for domestic and foreign scholars to study VRP. His idea was developed in 1975 by Professor Holland, a global research tool based on natural selection and genetic research. It stimulates the evolution of genes from genetic selection, crossover, and mutation and is used to determine the most efficient chromosomes using an energy function. Genetic algorithms modeled on Darwin's theory of natural evolution and genetic change are robust, globally optimized, and designed to solve complex multivariate optimization and combinatorial problems, and various forms of utility bills. The relative values are shown in Table 1 [11].

### 3.3. Genetic Algorithm Based on Multilogistic Center Collaborative Distribution Path Optimization

#### 3.3.1. Algorithm Description

*(1) Choose Code and Create a Population Default*. The program uses the natural copper encoding method. *m*_2_, *m*_4_, and *m*_6_ are distributors that randomly generate a large number of *n* users. 1,2,3 … *n* are all users. In this way, *m*_1_, *m*_2_, *m*_3_, 4, 5, 6, *n*_* *−1_, and *n*_*m*_ can be encoded to express the classification solution. They are *m*_1_ and 12m, which means that the distribution center distributes the goods to each household 1 and 2. Shipping to each distribution center must meet design constraints. Every operator can prove it by distributing it to its users. When selecting a distribution area for coding, we combine the previous distribution area with the next distribution area, convert *m*_1_ into *I*_2_, calculate the distribution area including *m*_13_ and *m*_33_, etc., and ensure that there is a user relationship between the two. According to this method, two adjacent parts get the first possible solution, the first group [12].

*(2) Fitness Function*. In order to evaluate the delivery process, the coding method is determined according to the specifics of the vehicle scheduling problem, i.e., each customer sends, each customer only delivers to one dealer, and the goods are sent to everyone. Machine constraints will not be satisfactory after multiple transfers. Therefore, the definition of the exercise function should reflect both the feasibility of the solution and the cost of the parallel process. For self *y*, the reciprocal of the relative distribution is *M*, 0M = 0, which means that this person is possible; if his goal is W, then the effective function of *y* is F, as described in equation (1).

(1) $$\begin{array}{c} y\frac{\sum_{r=1}^{p}\left(w_{y}+m_{y}\right)}{w+m_{p}}. \end{array}$$

Here, *p*_*ω*_ represents the severity of the failure (the weight can bring a good number for the maximum value of the target job). Combined with the actual policy, *p*_*ω*_ is 1000 [13].

### 3.4. Basic Theory of Logistic Distribution Path Problem

Ramser and Danzig originally proposed a vehicle routing problem (VRP) that solves a specific constraint (e.g., start time, end time, and transit time) and achieves a goal (e.g., shortest total number) within the maximum freight range (the shortest time, cost, vehicles, etc.)., which are essential for customers with various special transportation needs. Since the VRP problem is a complex problem in NP with similar production and environmental problems, the study of the VRP problem provides theoretical support for studying the problem of improving efficiency and effectiveness [14].

### 3.5. Application of Genetic Algorithm in VRP

#### 3.5.1. Modeling and Description of VRP Problems

The VRP (transportation issues) is one of the key issues in the modern logistic industry. This is also a difficult problem for NP and gradually becomes popular *n*. We combine cities and their points to find a system where each city is only passed once, and all goods are loaded on time. The explanation for the VRP problem is simple. In short, to find the shortest path or reduction value through *n* sending points, we find the plan for a subset of integers (*x* = *m*, where the elements of *n*_*X*_ represent the number of *n* cities) (*n*_*mX*_)2=*v*_*J*_. Equation *d*(*v*) represents the distance from city *v*_*i*_ to *v*_*i*+1_, as shown in equation (2).

(2) $$\begin{array}{c} T=\sum d\left(v_{i}+v_{i+1}\right)+d\left(v_{m}\right). \end{array}$$

#### 3.5.2. Genetic Operators for VRP

The VRP genetic operators usually include additional operators, crossover operators, exchange operators, and reverse operators [15].

*(1) Personnel Selection*. Common selection processes of VRP schemes include roulette wheel selection mechanism, optimal placement process, reliability model selection mechanism, category model selection mechanism, association selection mechanism, and isolation model. This form uses a roulette wheel to choose ideas. The main idea of the roulette selection mechanism is that the outcome of each candidate is proportional to the value of the energy function. If the population is *n* and the energy of an *i* is *F*_*i*_, then the probability of selecting *I* as the next generation heir is determined by equation (3).

(3) $$\begin{array}{c} p_{i}=F_{i}\sum_{i=2}^{m}F_{i}. \end{array}$$

*(2) Crossover Operator*. The cross operator is an important operator in the genetic algorithm, which gives a brief introduction to the more mature cross operator in the genetic algorithm. Common crossover operators have single-point crossover, double or multipoint crossover, uniform crossing, arithmetic crossing, etc., single-frequency division, digital frequency division, and the like. This recipe uses a partial cross recipe. Intersections with half a graph are also called partial intersections. All frequency division is divided into two stages: (1) two-point frequency division is required to operate on a single line of code; (2) according to the relationship diagram of different subvalues at the intersection, the sowing amount of different seeds outside the intersection area is adjusted [16].

*(3) Change in Business Operator*. The genetic algorithm describes the crossover operation. From a genetic algorithm perspective, solutions can only be developed through selection mechanisms and crossover strategies. Mutation simply fixes and adds some genes involved in the selection and hybridization process. Only change can understand the world of GA.

*(4) Operator Returns*. Operators have shown some growth in genetic work due to the shortcomings of early integration that often arise when using genetic processes to solve the VRP problems. Experiments show that the calculated optimal solutions can be seen in a wide range of applications. Therefore, well-thought-out algorithms can prevent early interactions and improve the global performance of genetic work [17].

### 3.6. Steps and Flowchart of Basic Genetic Algorithm

**Figure 2:**
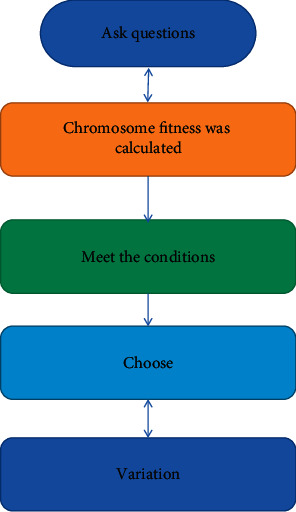
Flowchart of basic genetic algorithm.

The key steps are as follows: ① we determine the number of the first population, define the coding process, create the first population, and list all members of the first population as its citizens; ② the salary of each citizen will be calculated by rank, and it will be determined whether the operation is satisfied cost. If a good solution is encountered, if not, we go to step ③; ③ according to the cost and choice of the operator, we select the person who is most suitable for educating future generations; and ④ we create a new person in the community according to the cross-exchange operator and return to the step in the new population ②. The operation diagram is shown in Figure 2 [18].

## 4. Experiment and Research

### 4.1. Genetic Algorithm

The genetic algorithm is initiated by an arbitrarily initiated public, adopts a random selection mechanism, and is based on the principle of survival of the fittest in nature, so that very good people can have a lot of time to enter the future; newborns with strong adaptability are formed through overtime; and change ensures diversity. Over several generations of “evolution,” the population slowly reaches or remains normal until the algorithm converges. Through multiple operations, multiple chromosomes finally obtained positive results. The higher the cost of exercise, the better the drug. Therefore, the method of genetic coding is very high when using the genetic algorithm to optimize the vehicle logistic distribution network system. Chromosomes represent the solution to the LRIP problem. Chromosomes are coded for natural copper. Each chromosome consists of four parts, as shown in Table 2 [19].

#### 4.1.1. Model of Genetic Algorithm

**Figure 3:**
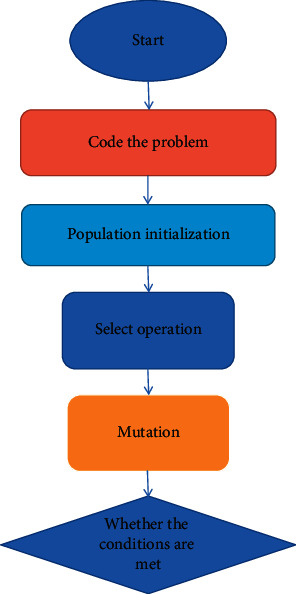
Work flowchart of genetic algorithm.

The genetic algorithm is an intelligent algorithm, which is often used as an important method to solve VRP (transportation problem). Professor Holland conducted the first international research on natural selection and genetics in 1975. He modeled the evolution of genetically engineered species, including selection, crossover, and transformation, and used force to demonstrate the efficiency of chromosomes. It has strong robustness and global optimization ability. It is necessary to solve complex multipole optimization problems and combinatorial problems, and has a wide application cost.  Figure 3 is a functional diagram of the genetic algorithm [20].

#### 4.1.2. Logistic Distribution Process

**Figure 4:**
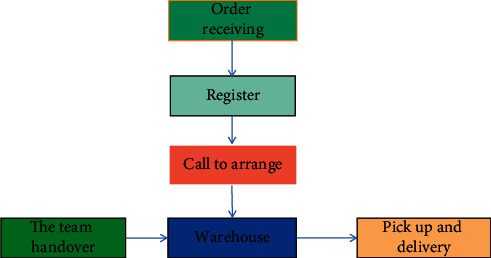
Flowchart of logistic distribution in local areas [21].

The normal operation process includes orders, registration, telephone, delivery, pick-and-place, in-transit, sign-in, and transportation, as shown in Figure 4.

In Figure 4, the received order is the main part of the shipment. If the shipper and the freight forwarder receive the shipping plan required by the customer, it affects the process; then, the shipment and transit time receive the consignor's cargo information, and finally, check and receive the consignor's waybill [22]. The registry is close to receiving linked orders. Its main purpose is to register the purpose of shipment and the delivery customer's bill of lading number and receipt received by your delivery person in the delivery area, and to confirm and sign the shipping register. Phone calls and shipping are the most important links in international shipping. The quality of the two links directly affects the performance of the logistic industry. The steps involved in making a call include planning for traffic, writing traffic information, submitting support services and tracking feedback, and completing forms using a computer. The shipper is the shipper who arranges the goods according to the final destination of the received goods, the weight of the goods, the volume of the goods, and the maximum carrying capacity of the vehicle, and informs the customers of their needs. Ensure the waiting time of vehicle arrival through reasonable transportation planning [23].

### 4.2. Division of Logistic Distribution Area

**Figure 5:**
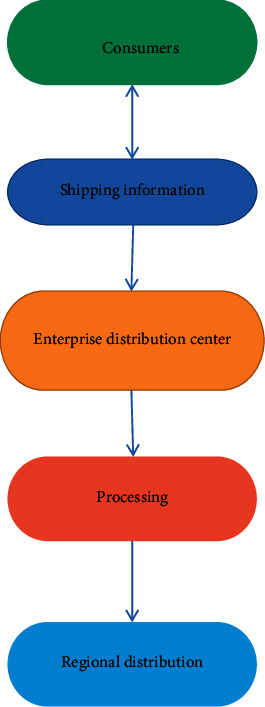
Route distribution diagram.

Before optimizing the logistic distribution path, we analyze the regional distribution process, as shown in Figure 5.

Based on the above analysis, the logistic distribution area is divided by the grouping algorithm. The distribution is as follows:

**Step 1 .**

We select the starting group, find *n* files in all data files in the group position, find *n* files in the file data of *n* = 1, 2,…, *n* information, and take *n* = 1, 2,…, *n* information that is based on group settings. According to the needs of the e-commerce logistic distribution center, the management of N groups of office partitions can be divided into (*x*_1_, *y*_1_), (*x*_2_, *y*_2_),…, (*x*_n_, *x*_n_) to ensure that the original distribution areas do not overlap.

**Step 2 .**

We use the distance model to find the distance between groups' center and each data. The calculation formula is shown in formula (4)as follows:

(4) $$\begin{array}{c} D\left(k+1\right)=\sqrt{g_{a}}-f_{a+1}. \end{array}$$

Among them, D(*K*+1) represents the distribution area; *g*_*a*_ represents the group center; and *f*_*a*+1_ represents the data of the group entity. According to the model, the center of the nearest data center is calculated and divided into centers. The goal is to save time for trucks on the road.

**Step 3 .**

We adjust the center position. Since the center of the *n* groups containing some of the most frequently used data changes, the data in the groups are modified according to the calculation results of step 2. It is calculated as follows: we divide the longitude coordinates of all points in a set by the number of points. According to this principle, the sum of all points is divided by the number of points to get the latitude and longitude coordinates of the new group and the location of the new group. The goal is to make the distribution as efficient as possible and avoid inconsistencies. Therefore, the volume of each particle must be equal.

**Step 4 .**

We determine the center position, use the center point count in step 3 for the next iteration cycle, and compare the group in the middle position count with the three steps above, and the middle position is final. The milk count is represented by formula (5)as follows:

(5) $$\begin{array}{c} K_{g=\sum_{a}q}•\frac{a}{f^{n}•h}. \end{array}$$

Among them, kg represents the product group of the logistic division; ∑*aq* represents the large group; *f*^*n*^ represents the distance from the group; *h* represents the measurement comparison group; and a represents the density of distribution points.

If the spatial positions calculated according to the model greatly differ, we recalculate so that the volume of each vehicle is equal.

### 4.3. Genetic Algorithm Logistic Distribution Path Optimization

**Figure 6:**
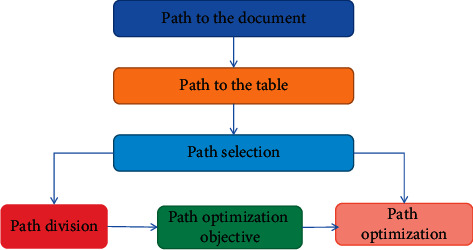
Path design process.

On the basis of the abovementioned logistic distribution area division, the logistic division method is optimized. We evaluate the design process prior to optimization. The design method is shown in Figure 6.

On this basis, the delivery method is established and optimized for various operational purposes, usually including weight measurement, time measurement, user value measurement, window measurement time, and total measurement methods. The special counting procedure is as follows. Weight index: in the process of regional logistics and distribution, the weight of the goods must be calculated, as shown in sections (6) and (7) for weights as follows:

(6) $$\begin{array}{c} S_{g}={\sum_{i}f\left(g-i\right)}^{tu}, \end{array}$$

(7) $$\begin{array}{c} Z_{i}=\frac{X_{i}}{X_{i}}. \end{array}$$

Among them, *S*_*g*_ represents the delivery place number; *f* represents the weight of the delivered goods; *g* is the regular source of logistic distribution; *i* is the weight of the goods shipped from the *i*th place; and cleanliness is a measure of weight.

Based on the above weight scale, the aging scale is calculated. As the e-commerce export market is more and more cold and new, and the requirements for delivery time are getting higher and higher, the aging index is designed to affect the demand for products in a timely manner. The formula is expressed as formulas (8) and (9)as follows:

(8) $$\begin{array}{c} S_{t}=\frac{1}{N}\left(\frac{t_{a}-t_{i}}{T_{fx}}\right), \end{array}$$

(9) $$\begin{array}{c} \left[\begin{array}{c} x_{1}=a_{1}F_{1}+a_{2}F_{2}+a_{1m}+F_{m}+\varepsilon \\ X_{2}=a_{2}F_{2}+a_{2}F_{2}+a_{2m}+F_{m}+\varepsilon_{2} \end{array}\right], \end{array}$$

where *t*_*i*_ represents the storage time of the goods at the *i*th distribution point; *T*_*fx*_ represents the time required to deliver the goods; *t*_*a*_ is the time to exit the distribution; 1/N represents the components involved in the distribution; and *S*_*t*_ is the estimated time of arrival of the product.

Finally, all measures for export logistics are included. Due to different logistic distribution methods, in order to improve distribution efficiency, we include the overall measures. The formula is shown in formula (10)as follows:

(10) $$\begin{array}{c} S_{k}=\frac{1}{F}\left(\frac{n+1}{h_{y}\cdot j}\right), \end{array}$$

where *S*_*k*_ represents the distance from the kth distribution point; 1/*F* represents the distance after shipment; *h*_*y*_·*j* is the exchange coefficient of the whole method; and *N* is the total value measurement.

Based on the above weights, a refinement of measurement time, customer value time, measurement time, and total measurement method, the delivery purpose is created. Since shipping companies charge different amounts for export purposes, the weight is set for distribution purposes. The above operational goals are not broad until set. The formula is expressed as formulas (11) and (12)as follows:

(11) $$\begin{array}{c} X^{e}=\frac{x-n}{M-m}, \end{array}$$

(12) $$\begin{array}{c} x=AF+\varepsilon , \end{array}$$

where *x*^*e*^ represents the value of the measurement in each input; *M* represents the minimum value in *m*; and *x* represents the maximum value in *n*.

On the basis of the above dimensionless processing, the weight is determined, and the relative importance is measured by comparing the two. The analysis is shown in Table 3.

According to the functions and weights of the above logistic distribution indicators, the distribution path is optimized, as shown in equation (13).

(13) $$\begin{array}{c} G=\eta *\left(W_{1},W_{2},W_{3},W_{4},W_{5}\right). \end{array}$$

They are as follows: *W*_1_, *W*_2_, *W*_3_, *W*_4_, and *W*_5_ represent the total weight of the weight indicator, time indicator, customer saliency indicator, time window indicator, and total traffic indicator, respectively; *η* is the proportional profit margin.

### 4.4. Test Data

The test data are provided by the logistic provider. A total of 7 items need to be delivered in the shipment from the fulfillment center. The main data of these seven projects are shown in Table 4.

According to the analysis in Table 4, the weights, values, and loading times of the seven distribution zones are different. The best way to find the seven products is to use the two methods, and the time to find the best way is compared with the two methods.

### 4.5. Analysis of Experimental Results

**Figure 7:**
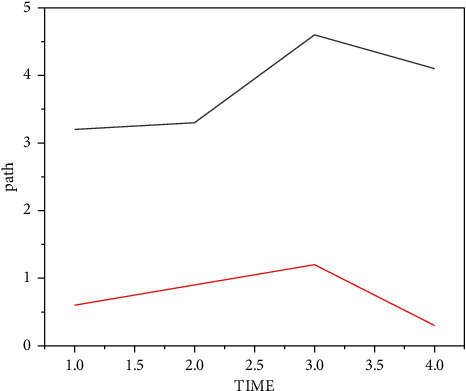
Comparison between optimization algorithm and traditional algorithm.

The time results of the logistic distribution path optimization algorithm and the traditional algorithm to find the optimal path are shown in Figure 7.

From the analysis in Figure 7, it can be seen that in this project, the time to find the best solution is less than 2 minutes, while in the normal process, the time to find the best method is more than 3 minutes. In contrast, the time required for traditional methods to find the optimal method is five times that of the algorithm developed this time.

From the above experiments, it can be demonstrated that the derived algorithm optimization method developed in this form in the context of big data has a shorter time to find a better method than the usual process and can meet the needs of good delivery.

To clearly demonstrate the effectiveness of the different algorithms, the best performance of the different algorithms is compared to the best method and the errors are tabulated. The results are shown in Table 5.

From the analysis in Table 5, it can be seen that among the two different optimization algorithms, the best way to derive the design time of the optimization algorithm is lower than the actual one, and each error is lower than the performance of the result, that is, the traditional algorithm. The above experimental results show that the best way to derive the optimization algorithm is to follow the best way, thus confirming the accuracy of designing the logistic distribution optimization algorithm.

## 5. Conclusions

This work has become increasingly important as the pace of global market integration accelerates, and as the technology develops rapidly, its share of the global market grows. It has gradually become an important indicator for domestic and foreign enterprises to measure their comprehensive competitiveness. Therefore, choosing the correct, appropriate, and effective research can save costs and improve the overall competitiveness of enterprises. Because the logistic distribution optimization process involves many disciplines and many influencing factors, it is necessary to use various algorithms to compare and identify different models. Classified logistics is an important part of the combinatorial optimization process and is robust. However, the optimization algorithm itself is rich in content and is in a stage of rapid development both in practice and theory.

## Figures and Tables

**Table 1:** Correspondence.

Biological genetic concept	Concepts expressed in optimization problems
Individual	Feasible solution
Chromosome	Solution coding
Gene	Characteristics of each component in connection
Individual fitness	Objective function of solution
Population	The set of feasible solutions

**Table 2:** Chromosome coding design.

1 bit	N-bit	K-bit	1 bit
Main engine factory	Vehicle number	Dealer service	Objective function value

**Table 3:** Relative column scale values.

Different levels of importance	Weight assignment
*i* and *j* two elements are equally important	2
Element *i* is slightly more important than element *j*	4
Element *i* is more important than element *j*	6
Element *i* is much more important than element *j*	8

**Table 4:** Logistic good information.

Serial number	Weight/kg	Importance/%	Unloading time/h
1	0.6	4	0.4
2	0.4	5	0.7
3	0.1	6	0.6
4	0.5	4	0.6
5	0.67	2	0.64
6	0.29	1	0.36
7	0.66	2	0.2

**Table 5:** Measurement error of travel path time.

Error index	Traditionalalgorithm	Algorithm inthis study
Relative error	95.20	77.03
Average relative error	9.54	7.44
Mean absolute relative error	7.84	5.98
Maximum absolute relative error	0.28	0.07
Square root of mean squaresum of relative error	0.07	0.02
Equalization coefficient	0.70	042

## Data Availability

The data used to support the findings of this study are available from the corresponding author upon request.
